# Ponatinib Is a Pan-BCR-ABL Kinase Inhibitor: MD Simulations and SIE Study

**DOI:** 10.1371/journal.pone.0078556

**Published:** 2013-11-13

**Authors:** Karunakar Tanneeru, Lalitha Guruprasad

**Affiliations:** School of Chemistry, University of Hyderabad, Hyderabad, India; University of Akron, United States of America

## Abstract

BCR-ABL kinase domain inhibition can be used to treat chronic myeloid leukemia. The inhibitors such as imatinib, dasatinib and nilotinib are effective drugs but are resistant to some BCR-ABL mutations. The pan-BCR-ABL kinase inhibitor ponatinib exhibits potent activity against native, T315I, and all other clinically relevant mutants, and showed better inhibition than the previously known inhibitors. We have studied the molecular dynamics simulations and calculated solvated interaction energies of native and fourteen mutant BCR-ABL kinases (M244V, G250E, Q252H, Y253F, Y253H, E255K, E255V, T315A, T315I, F317L, F317V, M351T, F359V and H396P) complexed with ponatinib. These studies revealed that the interactions between ponatinib and individual residues in BCR-ABL kinase are also affected due to the remote residue mutations. We report that some residues, Met244, Lys245, Gln252, Gly254, Leu370 and Leu298 do not undergo any conformational changes, while the fluctuations in residues from P-loop, β3-, β5- strands and αC- helix are mainly responsible for ponatinib binding to native and all mutant BCR-ABL kinases. Our work provides the molecular mechanisms of native and mutant BCR-ABL kinases inhibition by ponatinib at atomic level that has not been studied before.

## Introduction

The breakpoint cluster region-Abelson (BCR-ABL) is the cytoplasmic fusion oncoprotein with constitutive tyrosine kinase activity, associated with Philadelphia chromosome responsible for acute lymphoblastic and chronic myeloid leukemias [1]–[4]. Chronic myeloid leukemia (CML) is characterized by the reciprocal chromosomal translocation t (9;22) (q34;q11) that leads to produce the BCR-ABL [5]–[10]. Inhibitors of ABL kinase domain can be used to treat most chronic-phase of CML [11]. The drug resistance can be caused by amplification of the oncogenic protein kinase gene [12] or some other mechanisms. But in most cases, resistance can be traced to the selection of cancer cells with secondary mutations in the targeted kinase. These resistance mutations often appear in the kinase catalytic domain to weaken or prevent interactions with inhibitor [13]. The development of multiple generations of BCR-ABL kinase inhibitors serves as an important model for understanding and addressing resistance in other targets [14]. The ABL kinase inhibitor imatinib is effective drug with impressive response and survival rates in the chronic phase of disease [15], [16]. Though imatinib is most effective in many cases, mutations in BCR-ABL often lead to resistance. The cells get resistance to imatinib in the case of threonine to isoleucine mutation at position 315 (T315I) in active site and some other P-loop mutations [17]–[20]. The development of second-generation ABL inhibitors like nilotinib and dasatinib are active against many imatinib-resistant mutants [21]–[23]. Ponatinib (AP24534), a third generation pan-BCR-ABL kinase inhibitor generated from the structure-guided drug design strategy, is able to inhibit native BCR-ABL kinase, most of the clinically relevant mutants including T315I mutation [14].

Zhou et al., solved the crystal structure and made significant analysis of ponatinib in complex with native and ABL^T315I^ mutant kinases (PDB_IDs: 3OXZ and 3IK3) [14]. The crystal structures provide valuable information; the overall protein structures, the position of ponatinib and its interaction pattern with both native and mutant ABL^T315I^ kinases is highly similar. However, the crystal structure is a static and average structure that does not necessarily represent the true structure, where certainly the structure undergoes a rapid equilibrium within few conformations. Even though the crystal structures are closer to the structure *in vivo* or *in vitro*, possibly they differ significantly from the true structure; because experimental conditions of a crystal structure differ from real-life conditions. The mutational analysis from the static structure normally ignores short or long range conformational changes and they do not include the dynamic effects caused by thermal motions [24].

The molecular dynamics (MD) simulations and molecular mechanics-Poisson-Boltzmann surface area (MM-PBSA) calculations on the problem of imatinib resistance by various BCR-ABL mutations has been studied by Lee et al., [24]. Computational simulations can provide atomic level description of structural details, energy landscape, dynamic behaviours, and other properties which are difficult to be obtained from the experimental studies. Here, we report the MD simulations, solvated interaction energies (SIE) free energy calculations of ponatinib with native and mutants of BCR-ABL kinase. We have also calculated the contributions from individual amino acid residues in the active site of all complexes to provide the molecular basis for inhibition. To our knowledge these studies have not been carried out before and our results provide detailed information about the molecular mechanisms of inhibition of native and various mutant BCR-ABL tyrosine kinases when bound to ponatinib.

## Materials and Methods

The 3D crystal structure of ABL kinase domain complexed with ponatinib was used as the initial structure (PDB_ID: 3OXZ) [14]. In this structure, the kinase domain spans the region from 242–493 amino acid residues. All crystal water molecules were removed and the missing amino acid residues in the structure were built based on its protein sequence using Discovery Studio 2.1 (D.S 2.1; Accelrys Software Inc., San Diego, CA). Single amino acid mutations were incorporated in ABL kinase using “protein modeling” protocol, “build mutation” module in D.S 2.1 and the structures were optimized using D.S 2.1. All MD simulations were performed using GROMACS 4.5.4 package [25], [26] with Amber ff99SB force field [27]. The ligand parameter files with Amber ff99SB and GAFF force fields using antechamber [28], [29] were generated by using ACPYPE script [30]. The complexes were subjected to MD simulations for 25 nano seconds (ns) at isothermal-isobaric conditions in a periodic cubic box with an edge length of approximately 10 Å. The protein was solvated in the cubic box using explicit solvent- SPC/E model water molecules around protein complex and its charge was neutralized using sodium ions. The native and mutant systems were neutralized; E255K with 10 Na^+^, E255V with 11 Na^+^, G250E with 13 Na^+^, and remaining mutants were neutralized with 12 Na^+^ ions. During the MD simulations, we initially performed 5000 steps of steepest descent minimization and 1000 pico seconds (ps) position restrained dynamics to distribute water molecules throughout the system. Finally we performed MD simulations of the whole system for 25 ns, using 0.002 ps time step. The Particle Mesh Ewald (PME) summation method [31], [32] was employed for the calculation of electrostatics, with a real space cut-off of 10 Å, PME order of 6 and a relative tolerance between long and short range energies of 10^−6^. Short range interactions were evaluated using a neighbour list of 10 Å updated every 10 steps, and the Lennard-Jones (LJ) interactions and the real space electrostatic interactions were truncated at 9 Å. The V-rescale thermostat [33] was used to maintain the temperature; the Parrinello-Rahman algorithm [34] was employed to maintain the pressure at 1 atm and hydrogen bonds were constrained using LINCS algorithm [35]. The trajectory file obtained from MD simulations was used for calculation of free energy of binding and other analysis. The RMSD (root mean square deviation) of certain atoms in a molecule with respect to a reference structure can be calculated with the program g_rms of GROMACS by least-square fitting the structure to the reference structure. The hydrogen bonding interactions between ponatinib and ABL kinase were calculated using program g_hbond of GROMACS.

### Solvated interaction energies

The SIE values were calculated using parameters that have been fitted to reproduce binding free energies for a data set of 99 protein-ligand complexes [36], [37]. It is an end-point physics-based, force-field-based scoring function for predicting ligand-binding affinities. This approximation to binding free energy in solution resembles the formalism used in other binding free energy end-point calculation methods, including MM-PB (GB)/SA [38]–[41] and Linear Interaction Energy (LIE) [42]. Binding free energies (ΔG) for all protein-ponatinib complexes were estimated using sietraj program [36], [43]. Sietraj (http://www.bri.nrc.ca/ccb/pub) is a set of scripts and executables for carrying out the SIE calculation on a MD trajectory or single snapshot of a target-ligand complex. This program calculates ΔG for snapshot structures from MD simulation with a rigid infinite separation of protein and ligand [36]. The ΔG is a sum of intermolecular van der Waals (vdW) and coulomb interactions plus change in the reaction field energy (determined by solving the Poisson–Boltzmann equation) and non-polar solvation energy (proportional to the solvent-accessible surface area) [36]. Sietraj is an alternative to the MM-PBSA software provided by AMBER distribution and SIE treats the protein-ligand system in atomistic detail and solvation effects implicitly. The free energy of binding between inhibitor and protein is computed by:



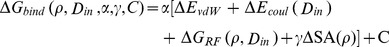
(1)Where ΔE_vdW_ and ΔE_Coul_ are the intermolecular vdW and coulomb interaction energies between protein and ligand, ΔG_RF_ (ρ,D_in_) is the difference in reaction-field energy between bound and free state of protein-ligand complex as calculated by solving Poisson equation with BRIBEM [44]–[46]. The term ΔSA (ρ) is the difference in molecular surface area between bound and free state of protein and cavity energy is change in the molecular surface area ΔSA that is calculated from γΔSA (ρ). The default values of parameters are: ρ = 1.1, D_in_  = 2.25, γ = 0.0129 kcal/(mol^3^ A^2^), C = −2.89 kcal/mol, and α = 0.1048. The ΔG is then scaled by an empirically determined factor and the five parameters in eq. 1 were fitted to the obtained absolute ΔG by fitting to a training set of 99 protein-ligand complexes [36]. The linear scaling factor ρ is the vdW radii of AMBER99 force field, D_in_ is the solute interior dielectric constant, the coefficient γ is molecular surface tension coefficient describing the non-polar component of solvation free energy, and the prefactor α implicitly quantifies the loss of entropy upon binding, also known as entropy-enthalpy compensation, and a constant C that includes protein-dependent contributions not explicitly modeled by SIE methodology, i.e., the change in protein internal energy upon ligand binding. The scaling can be considered as a crude treatment of entropy–enthalpy compensation containing the caveats of implicit solvation and neglecting the vibrational entropy [36], [47]. Here, we estimated ΔG averaging 250 structures from 25 ns of selected MD snapshots and averaging over the resulting free energies obtained from each snapshot. The individual residue contributions could help in estimating the corresponding amino acid effects on drug binding and also in the studies of drug resistance problems. Using sietraj program, we have calculated each amino acid residue contribution from electrostatic and vdW energies as the components of free energy binding of ponatinib to native and mutant BCR-ABL kinases.

The ineffective pairs of protein-inhibitor complexes were studied using imatinib and dasatinib complexed with BCR-ABL^T315I^. For these studies, we have used similar conditions of MD simulations and analyses described above.

## Results and Discussion

**Figure 1:**
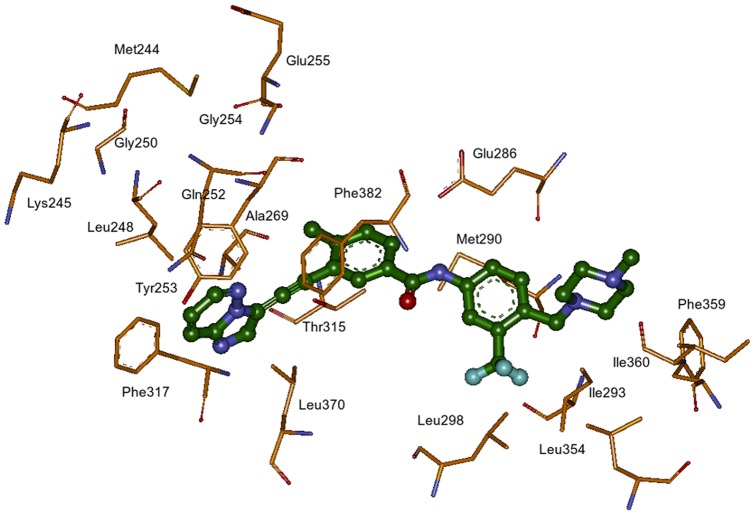
The amino acid residues of BCR-ABL kinase in the ponatinib binding site. Amino acid side chain carbons are shown in orange and ponatinib carbons are show in green; oxygen-red; nitrogen-blue; fluorine-cyan.

**Figure 2:**
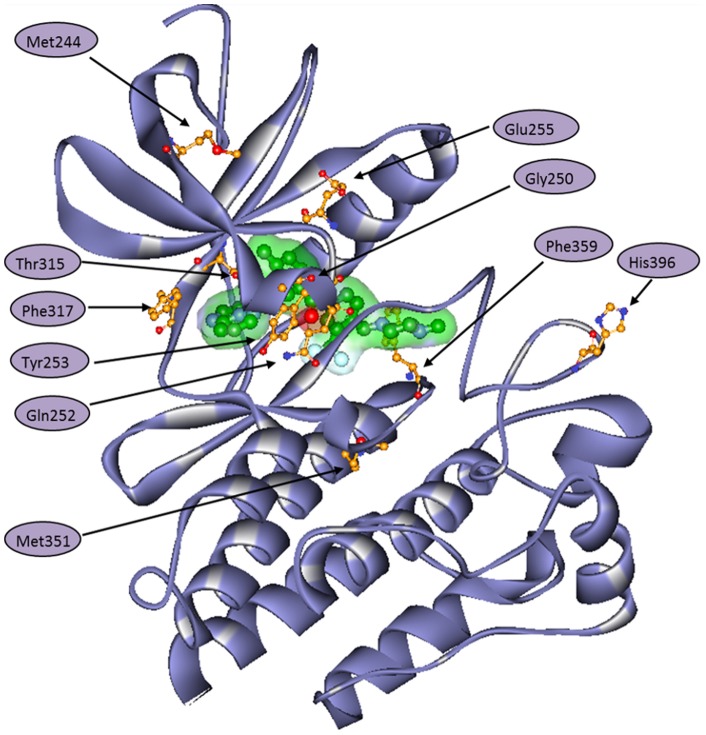
3D schematic representation of BCR-ABL kinase (purple) with most frequent mutations shown as ball and stick models with atom color representation: orange-carbon, red-oxygen, blue-nitrogen and yellow-sulphur. Ponatinib in the active site is shown as solvent accessible surface (green) covered ball and stick model.

The native and mutant ABL kinase – ponatinib complexes with explicit water molecules and sodium ions for charge neutralization were subjected to 25 ns MD simulations. The fourteen BCR-ABL mutants studied in this work collectively represent more than 95% of clinically observed mutations that are imatinib resistant. With the exception of T315I, most BCR-ABL mutations are inhibited by dasatinib and nilotinib. Ponatinib inhibits native and all mutant ABL kinases with high affinity [14], although some mutants have slightly greater inhibition than the others. The ATP competitive inhibitors of ABL kinase are classified into DFG-in or DFG-out classes depending on their binding interactions with kinase domain. Ponatinib binds to ABL kinase domain with a DFG-out conformation (PDB_ID: 3OXZ and 3IK3) and serves to distribute binding energy over a wide range of amino acid residues in the active site as shown in Figure 1. The presence of such optimized and distributed binding interactions has the potential to allow ponatinib to withstand modest reduction in potency caused by single mutation. For our convenience; we grouped these mutations by the region of their location in ABL kinase structure. These regions include the P-loop mutants M244V, G250E, Q252H, Y253F, Y253H, E255K, and E255V; gatekeeper residue mutants T315A and T315I; hinge region mutants F317L and F317V; activation loop mutant H396P and other mutants M351T and F359V. The location of mutations in BCR-ABL kinase is shown in Figure 2.

In the ABL kinase, amino acid residues Tyr253, Thr315, Phe317 and Phe359 are located in close contact with ponatinib and therefore affect the binding and activity of inhibitor. The P-loop mutant residues Gly250, Gln252 and Glu255 are not in direct contact with ponatinib, but share non-bonding interactions with inhibitor. The rest of the mutations Met244, Met351 and His396 are located away from inhibitor binding site, but intriguingly display ponatinib based inhibition.

### Molecular dynamics simulations and SIE binding free energy

**Figure 3:**
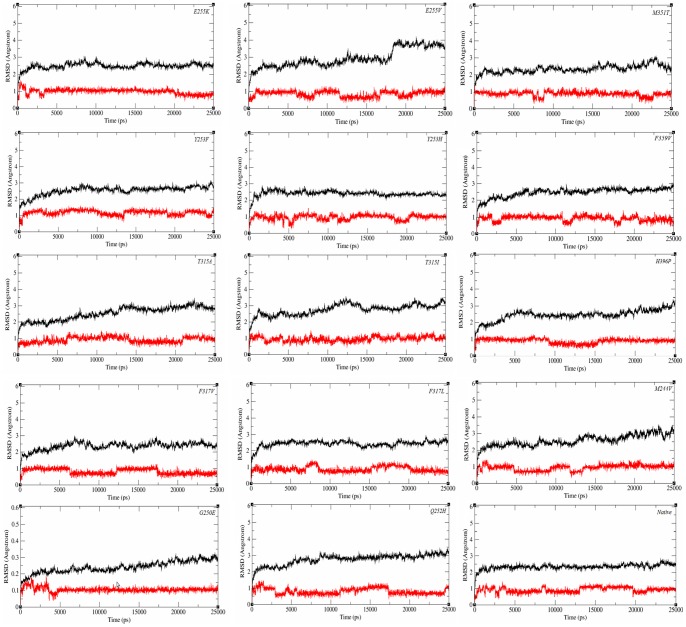
The RMSD plots of the native and mutant BCR-ABL kinases (black) complexed with ponatinib (red).

The 3D structures of native and mutant ABL kinases complexed with ponatinib were subjected to MD simulations to find optimal interactions and molecular basis for binding. Examination of the residue-wise RMSD showed that C-terminal part of the non-kinase region (501–511) had high variability. In some BCR-ABL 3D structures this C-terminal region is missing (PDB_ID: 2V7A, 3QRJ) [48], [49] or was shown to have variable conformations (PDB_ID: 1OPK, 3K5V) [50], [51]. Hence for analysis of MD results in this work, we deleted the highly fluctuating non-kinase region from 501–511 amino acid residues in BCR-ABL 3D structure (PDB_ID: 3OXZ). The RMSD plots for ponatinib complexed with various ABL mutants shown in Figure 3 revealed that protein structures finally converged to less than 4 Å RMSD and ponatinib had nearly 1 Å RMSD. These plots show that ponatinib remains bound to native and mutant ABL kinases near the preferential binding position and experiences fewer fluctuations during MD simulations with respect to its initial position.

SIE calculations from MD trajectories measure the free energy of complex formation. Table 1 shows the calculated free energies for native and 14 mutant BCR-ABL – ponatinib complexes. The intermolecular vdW, intermolecular coulomb and change in surface area are shown in Table 1. This table indicates that IC50 values [14] vary from 0.5 nM to 36 nM and SIE values calculated from this work are in the range −10.03 kcal/mol to −10.67 kcal/mol. Though there is no direct correlation between IC50 and SIE values, it can be observed that their respective values lie within a narrow range.

**Table 1:** List of ponatinib inhibition (IC50), binding free energies with native and mutant BCR-ABL kinases.

	IC50	vdW	Coul	RF	Cavity		ΔG
Mutant	(nM)	(kcal/mol)	(kcal/mol)	(kcal/mol)	(kcal/mol)	Const	(kcal/mol)
Native	0.5	−69.67	−6.17	16.21	−12.16	−2.89	−10.41
M244V	2.2	−70.71	−5.34	15.11	−12.21	−2.89	−10.55
G250E	4.1	−69.8	−6.26	16.19	−12.14	−2.89	−10.43
Q252H	2.2	−69.62	−5.87	16.64	−12.41	−2.89	−10.36
Y253F	2.8	−68.12	−5.63	18.15	−12.59	−2.89	−10.03
Y253H	6.2	−69.66	−5.77	17.01	−12.44	−2.89	−10.31
E255K	14	−70.74	−4.64	13.46	−12.37	−2.89	−10.67
E255V	36	−67.75	−5.46	15.23	−12.34	−2.89	−10.26
T315A	1.6	−69.74	−6.22	16.36	−12.45	−2.89	−10.44
T315I	11	−68.6	−7.01	16.57	−12.19	−2.89	−10.35
F317L	1.1	−68.52	−6.75	16.72	−12.17	−2.89	−10.3
F317V	10	−68.89	−5.69	16.4	−12.38	−2.89	−10.28
M351T	1.5	−69.47	−5.85	15.68	−12.19	−2.89	−10.41
F359V	10	−69.53	−5.78	16.29	−11.97	−2.89	−10.33
H396P	1.1	−68.93	−6.19	16.77	−12.32	−2.89	−10.29

The SIE binding free energy (ΔG) and its components van der Waal – vdW; Coulomb interaction – Coul; Reaction Field-RF.

Many patients eventually developed imatinib resistance, usually associated with above mentioned mutations in ABL kinase domain that either directly or indirectly effects the binding affinity of imatinib to ABL [12], [52]. The most common gatekeeper residue mutation T315I that accounts for 15–20% of clinically observed mutations is completely resistant to imatinib, nilotinib and dasatinib [49]. Native and T315I BCR-ABL kinases complexed with dasatinib are subjected to 25 ns of MD simulations and SIE binding free energies are calculated. The analysis of dasatinib complexed with native and T315I mutant BCR-ABL kinases revealed that native complex has relatively higher SIE free energy (−9.53 kcal/mol) than when complexed with T315I (−8.44 kcal/mol) that signifies the greater affinity of dasatinib for native compared to mutant BCR-ABL kinase.

The RMSD of BCR-ABL kinase – ponatinib complexes shown in Figure 3 indicated that in the native complex, ABL kinase converged from 2.5 ns of MD and ponatinib converged from 1 ns to the end of simulations. The SIE calculated free energy for native complex is −10.41 kcal/mol. The gatekeeper mutant T315I has a longer side chain and the less common gatekeeper mutant T315A has a smaller side chain when compared to Thr315. The calculated free energies correlate with experimentally measured IC50 values and comparably ponatinib has better binding towards the mutation T315A (−10.44 kcal/mol) than T315I (−10.35 kcal/mol). The free energy of BCR-ABL^T315I^ complexed with imatinib is −9.89 kcal/mol indicating that ponatinib has higher binding towards T315I mutation compared to imatinib.

Table 1 shows the distribution of electrostatic potential and contribution from neighbouring residues during MD simulations that are responsible for this free energy change. The mutation Y253F and Y253H present on the P-loop is in close contact with imidazo [1,2b] pyridazine of ponatinib. The Y253F mutation has 2 fold greater activity than Y253H [14], although the net SIE values for both complexes do not correlate with observed experimental values. These mutations show decrease in intermolecular coulomb energies (Y253F is −5.63 kcal/mol and Y253H is −5.77 kcal/mol) compared to native kinase (intermolecular coulomb value −6.17 kcal/mol) and Y253F mutation shows decreased vdW interaction energies (−68.12 kcal/mol). Phe317 located at middle of the hinge region is in ATP binding site, imidazo [1,2b] pyridazine ring of ponatinib interacts with Phe317 via pi-pi stacking and vdW contacts. From the analysis of MD simulations of F317V BCR-ABL kinase – ponatinib complex, we observed slightly increased intermolecular vdW energy (−68.89 kcal/mol) and cavity value (−12.38 kcal/mol) and decreased intermolecular coulomb value (−5.69 kcal/mol). The SIE free energy for F317V BCR-ABL kinase – ponatinib complex is (−10.28 kcal/mol) which is close to the SIE free energy of F317L (−10.30 kcal/mol).

The residue Phe359 is located on turn region at the end of αC- helix and is involved in the formation of a hydrophobic core with several residues from αC- helix including hydrophobic amino acids Val289 and Ile293. The F359V is adjacent to piperazine solubilization group of ponatinib and forms weak vdW interactions. The SIE binding free energy (−10.33 kcal/mol) was observed for this complex. In spite of side chains being oriented away from the binding site of ponatinib, the P-loop mutations E255K and E255V are closer to ponatinib effecting its activity. The residues G250E and Q252H are also present on the P-loop region but are not in direct contact to affect ponatinib binding. The remaining three mutants (M351T, H396P and M244V) in ABL kinase structure are located away from ponatinib binding site but are inhibited by ponatinib. The binding free energies calculated from SIE indicated that these mutants may be involved in the long range interactions with ponatinib. The free energies and reaction field energies from Table 1 explain the important contributions from these mutant residues.

### Intermolecular hydrogen bonding

**Figure 4:**
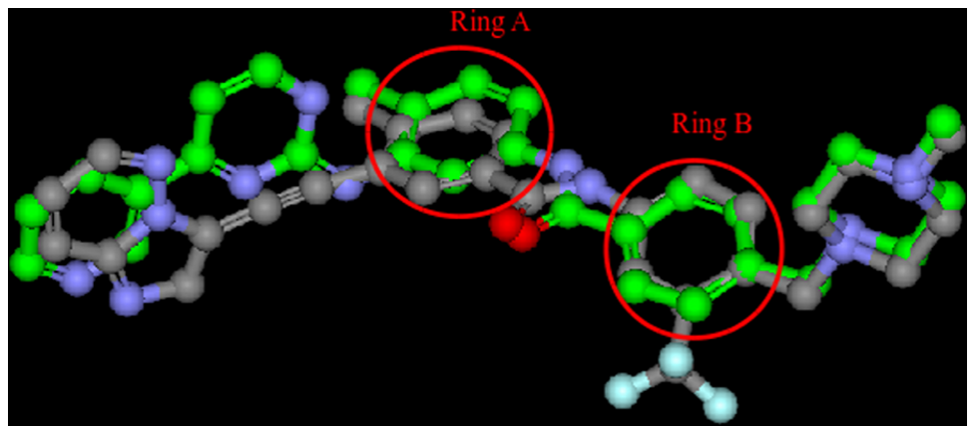
3D structural alignment of the imatinib (green) and ponatinib (gray) and the ring A and ring B are shown in red colour circles.

The hydrogen bonding interactions between ponatinib with various mutants in ABL kinase domain also explain the binding affinity. A maximum of three hydrogen bonds are observed between protein and ponatinib. From the crystal structure of ponatinib bound ABL kinase, imidazo [1,2b] pyridazine ring of ponatinib nitrogen forms hydrogen bond with main chain NH of Met318. This hydrogen bond remained constant throughout 25 ns of MD simulation for native and all fourteen mutant complexes. The amide linker between ring A and ring B (Figure 4) of ponatinib forms two hydrogen bonds with residues in kinase ATP binding site. From the crystal structure, carbonyl group of the amide linker forms hydrogen bond with main chain NH of Asp381 from DFG motif and NH of the amide forms hydrogen bond with side chain of Glu286 on the αC- helix, although these hydrogen bonds exchange with some other hydrogen bonds during MD simulations. The number of hydrogen bonding interactions in each of the complexes is shown in Figure S1.

### Energy contribution from individual active site residues in native and mutant BCR-ABL – ponatinib complexes

The free energy binding changes of protein – inhibitor complex with mutation provides an overall estimate of the increased or decreased affinity in complex formation. In this work, we observed that SIE free energy of binding does not vary appreciably in all complexes which are indicative that ponatinib is an effective inhibitor of native and all mutant BCR-ABL kinases which is in agreement with the experimental results [14]. Therefore, in order to decipher the contributions from each residue in ponatinib binding region, we have deconvoluted the energy contributions for interactions between ponatinib and BCR-ABL kinase into electrostatic and vdW type. The electrostatic part represents columbic interactions that include charge-charge and other multipole interactions. The vdW part is van der Waals forces of attraction or contact energy term. These two terms represent major part of non-bonding interactions in MD simulations [24]. In general terms, a positive value indicates a reduction in binding energy where as negative energy indicates that the binding is stronger.

The values of energy contribution from individual amino acid residues in ponatinib binding pocket are shown in Table 2 and the active site amino acid residues with ponatinib are shown in Figure 1. The mutations F317V, Y253F, Y253H and E255K experience increased vdW interactions with residue Leu248. The mutations E255K, F317L and F317V experience increased electrostatic interactions with Tyr253. Nearly all mutations experience variable vdW interactions ranging from −0.99 to −2.15 kcal/mol with Tyr253 indicating variable conformations adopted by the side chain owing to mutations but still retains the binding to ponatinib. Similarly, all mutations experience variable vdW interactions ranging from −2.96 to −3.82 kcal/mol with Phe382 and −2.13 to −3.46 kcal/mol with Phe359. The mutations T315I, E255V, F317L and F317V contribute to decreased vdW interactions with Ala269. The mutations E255K and T315A experience increased vdW interactions with Leu354. All mutations experience variations in electrostatic interactions ranging from −4.95 to −6.71 kcal/mol with Glu286, in particular, Q252H, Y253H, T315I and F317L experience increased electrostatic interactions with Glu286. The mutations E255V, M244V, T315A, Y253H, experience increased vdW interactions with Met290 and the mutation M244V experiences increased vdW interactions with Ile293. The mutations G250E, Y253H and T315I experience decreased vdW interactions with Phe359. The mutations Y253F, E255V and H396P experience decreased vdW interactions with Phe382. The mutation Y253H experiences decreased vdW interactions with Phe317, while structurally these two side chains are located on either side of imidazo [1,2b] pyridazine ring.

**Table 2:** The contribution from van der Waals (vdW) and coulombic (Coul) interaction energies to SIE binding free energy of ponatinib for different mutants from selected binding site residues.

Residue	Native		M244V		G250E		Q252H		Y253H		Y253F		E255K		E255V		T315A		T315I		F317V		F317L		M351T		F359V		H396P	
	vdW	Coul	vdW	Coul	vdW	Coul	vdW	Coul	vdW	Coul	vdW	Coul	vdW	Coul	vdW	Coul	vdW	Coul	vdW	Coul	vdW	Coul	vdW	Coul	vdW	Coul	vdW	Coul	vdW	Coul
M244	−0.02	0	−0.02	0.02	−0.03	−0.01	−0.03	0	−0.02	0.01	−0.03	0	−0.03	0.01	−0.02	−0.01	−0.02	0	−0.02	0	−0.02	0.01	−0.03	0	−0.03	0	−0.03	0	−0.03	0
K245	−0.02	0.26	−0.02	0.25	−0.02	0.22	−0.02	0.25	−0.02	0.26	−0.02	0.26	−0.02	0.24	−0.02	0.21	−0.02	0.21	−0.02	0.18	−0.02	0.2	−0.02	0.23	−0.02	0.24	−0.02	0.25	−0.02	0.21
L248	−2.11	0.16	−2.14	0.12	−2.36	0.05	−2.39	0.02	−2.46	0.12	−2.63	0.05	−2.71	0.25	−2.29	0.09	−2.13	0.1	−2.14	0.12	−3.07	−0.03	−2.33	0.02	−2	0.23	−2.06	0.07	−2.07	0.19
G250	−0.02	0.03	−0.02	0.02	−0.03	−0.32	−0.02	0	−0.03	0.03	−0.02	0.01	−2.67	−5.46	−0.02	0.02	−0.02	0.02	−0.02	0.02	−0.03	0	−0.02	0.01	−0.02	0.02	−0.02	0.02	−0.02	0.01
Q252	−0.11	−0.04	−0.13	−0.01	−0.13	0.03	−0.12	0.12	−0.13	0.23	−0.1	0.02	−0.03	0.02	−0.13	0.07	−0.12	0.03	−0.13	0	−0.11	0.08	−0.14	0.02	−0.13	0.01	−0.12	0	−0.13	0.03
Y253	−1.9	0.22	−2.05	−0.06	−1.96	0.01	−1.97	−0.03	−1.31	−0.3	−1.74	−0.22	−0.99	−0.12	−1.92	−0.02	−2.15	−0.02	−2.14	−0.08	−1.93	−0.29	−1.89	−0.11	−2.12	−0.03	−2	0.08	−2.1	−0.04
G254	−0.05	0.04	−0.06	0.04	−0.05	0.05	−0.07	0.04	−0.06	0	−0.05	0.03	−0.05	0.01	−0.05	0.02	−0.05	0.03	−0.07	0.05	−0.05	0.03	−0.05	0.04	−0.06	0.04	−0.05	0.04	−0.06	0.04
E255	−0.1	−0.59	−0.1	−0.5	−0.1	−0.57	−0.11	−0.51	−0.1	−0.54	−0.11	−0.54	−0.12	0.35	−0.11	−0.09	−0.09	−0.49	−0.1	−0.55	−0.09	−0.52	−0.09	−0.49	−0.1	−0.5	−0.09	−0.56	−0.1	−0.47
A269	−2.56	−0.18	−2.41	−0.19	−2.28	−0.11	−2.24	−0.14	−2.24	−0.19	−2.25	−0.12	−2.3	−0.16	−2.09	−0.15	−2.55	−0.24	−2.12	−0.13	−2.11	−0.12	−1.95	−0.11	−2.35	−0.17	−2.64	−0.2	−2.25	−0.18
E286	−2.66	−5.29	−2.91	−5.23	−2.61	−5.43	−2.66	−5.97	−2.44	−6.52	−2.55	−4.95	−2.67	−5.46	−2.75	−5.14	−2.76	−5.51	−2.73	−6.71	−2.6	−5.11	−2.73	−5.79	−2.7	−5.27	−2.74	−5.32	−2.63	−5.52
M290	−3.96	−0.71	−4.54	−0.64	−4.13	−0.66	−4.01	−0.64	−4.25	−0.57	−4.21	−0.74	−4.08	−0.71	−4.29	−0.67	−4.28	−0.67	−4.23	−0.51	−4.1	−0.76	−4.15	−0.68	−4.02	−0.76	−4.22	−0.7	−4.02	−0.75
I293	−1.73	−0.08	−2.57	0.07	−1.73	−0.03	−1.6	−0.09	−2.1	0.16	−1.81	−0.09	−1.63	−0.07	−1.6	−0.06	−1.86	0.06	−1.82	0.01	−1.71	−0.07	−1.7	−0.08	−1.64	−0.08	−1.88	0.09	−1.75	−0.06
L298	−1.17	−0.5	−1.11	−0.45	−1.34	−0.5	−1.22	−0.49	−1.02	−0.48	−1.15	−0.46	−1.23	−0.49	−1.15	−0.49	−1.37	−0.49	−1.23	−0.46	−1.31	−0.51	−1.2	−0.48	−1.12	−0.46	−1.32	−0.51	−1.29	−0.48
T315	−3.37	−0.27	−3.31	0.05	−3.42	−0.06	−3.26	0.05	−3.18	0.09	−3.42	−0.09	−3.13	0.01	−3.3	0.01	−2.05	−0.09	−4.11	−0.04	−3.26	0.03	−3.2	0.07	−3.35	0.1	−3.43	−0.02	−3.49	0.03
F317	−2.64	−0.58	−2.58	−0.48	−2.55	−0.35	−2.45	−0.35	−2.09	−0.36	−2.37	−0.34	−2.34	−0.39	−2.47	−0.48	−2.62	−0.50	−2.38	−0.53	−1.65	−0.40	−2.09	−0.54	−2.60	−0.44	−2.69	−0.62	−2.59	−0.44
L354	−1.29	−0.2	−1.49	−0.2	−1.5	−0.22	−1.42	−0.23	−1.14	−0.19	−1.16	−0.19	−1.63	−0.23	−1.43	−0.23	−1.81	−0.2	−1.49	−0.22	−1.5	−0.23	−1.54	−0.21	−1.19	−0.21	−1.25	−0.19	−1.41	−0.21
F359	−3.35	−0.5	−3.33	−0.42	−2.86	−0.44	−3.12	−0.47	−2.41	−0.32	−3.46	−0.43	−3.04	−0.43	−3.11	−0.46	−3.18	−0.53	−2.93	−0.46	−3.13	−0.51	−3.38	−0.52	−3.43	−0.5	−2.13	−0.16	−3.25	−0.54
I360	−1.31	0.18	−1.1	0.36	−1.3	0.25	−1.11	0.3	−1.26	0.37	−1.1	0.31	−1.11	0.34	−1.26	0.32	−1.21	0.24	−1.34	0.27	−1.18	0.31	−1.28	0.25	−1.25	0.3	−1.11	0.38	−1.22	0.28
L370	−3.57	−0.04	−3.48	−0.05	−3.51	−0.04	−3.42	−0.06	−3.70	−0.06	−3.43	−0.05	−3.62	−0.06	−3.49	−0.04	−3.45	−0.04	−3.43	−0.04	−3.35	−0.04	−3.52	−0.04	−3.53	−0.04	−3.44	−0.02	−3.41	−0.03
F382	−3.63	−0.3	−3.57	−0.31	−3.36	−0.34	−3.31	−0.38	−3.75	−0.31	−3.11	−0.35	−3.63	−0.32	−2.96	−0.38	−3.44	−0.36	−3.82	−0.4	−3.52	−0.4	−3.34	−0.37	−3.52	−0.35	−3.52	−0.36	−3.17	−0.38

The vdW and coulomb energies are calculated in kcal/mol.

The T315I mutation experiences increased (−4.11 kcal/mol) and T315A mutation experiences decreased electrostatic interactions (−2.05 kcal/mol), and the mutation G250E contributes increased electrostatic interaction (−0.32 kcal/mol) while binding to ponatinib. The mutation F359V experiences decreased vdW (−2.13 kcal/mol) and electrostatic interactions (−0.16 kcal/mol), while decreased electrostatic interactions are experienced due to mutations E255K (0.35 kcal/mol) and E255V (−0.09 kcal/mol). The mutation Y253F (−0.22 kcal/mol) experiences increased electrostatic interactions, Y253H experiences increased electrostatic interactions (−0.3 kcal/mol) and decreased vdW (−1.31 kcal/mol) when binding to ponatinib. The F317 mutation experiences decreased electrostatic interactions −1.65 kcal/mol and −2.09 kcal/mol due to mutations F317V and F317L respectively.

From these results we observed that the P-loop residues Leu248 and Tyr253 that closely interact with ponatinib undergo highly fluctuating conformational changes affecting the contribution from some residues in active site. In an analogous manner, the active site residue Ala269 located on the β3- strand from N- terminal domain also has conformational changes while interacting with ponatinib. The amino acid Glu286 from the αC- helix also has variable electrostatic interactions due to mutations in BCR-ABL kinase domain. The Phe359 caps ponatinib binding site close to the activation loop and undergoes conformational changes with most mutations in particular, the P-loop residues (G250E and Y253H) and the gatekeeper mutation T315I on the β5- strand. The Phe382 from the DFG motif undergoes conformational changes with most mutations in particular; the P-loop residues (Y253F and E255V) and the activation loop residue H396P. The fluctuations in residues from P-loop, β3-, β5- strands and αC- helix are mainly responsible for ponatinib binding to native and all mutant BCR-ABL kinases.

Lee *et al*., proposed that T315I mutation leads to significant conformational changes and therefore a decrease in the binding affinity between imatinib and ABL kinase domain. Importantly, the αC- helix is flexible and residues Glu286 and Met290 located at ligand entrance site show reduced interactions with T315I. Also decrease in electrostatic interactions for E255, Q252 and Y253 are responsible for imatinib resistance [24]. In this study of ponatinib binding to native and mutant BCR-ABL kinases, we have also observed high fluctuations of Glu286 and Met290 amino acid residues while binding to ponatinib, although the effect of Met290 is lower compared to Glu286. Similarly, Tyr253 undergoes high conformational changes due to mutations while no significant alterations were observed with Glu255 and Gln252.

**Figure 5:**
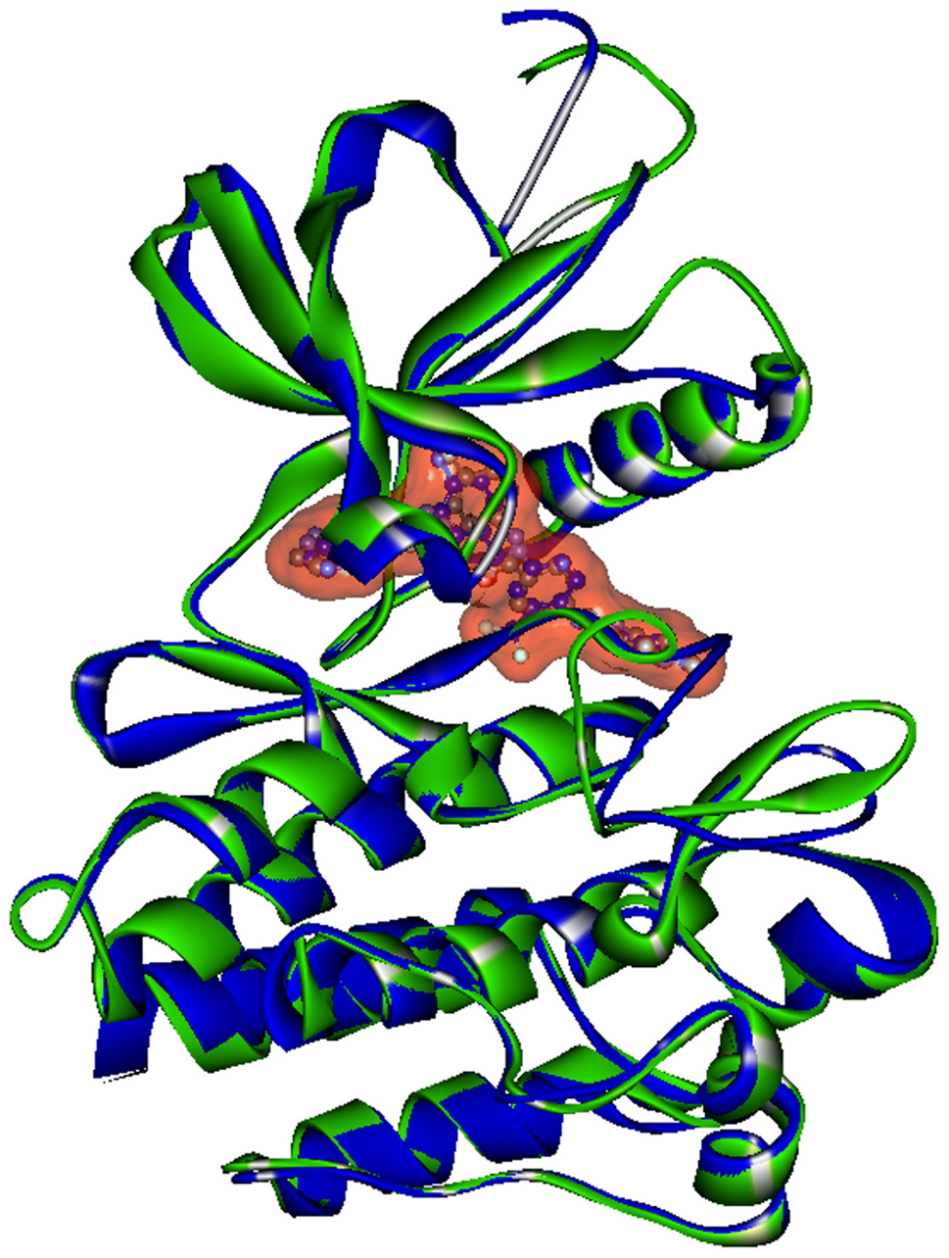
Structure alignment of imatinib bound BCR-ABL kinase (PDB_ID: 1IEP) (green) and ponatinib bound BCR-ABL kinase (PDB_ID: 3OXZ) (blue). The imatinib and ponatinib are represented in ball and stick models covered by solvent accessible surface (orange).

The crystal structures of BCR-ABL kinase bound to imatinib (1IEP_A) and ponatinib (3OXZ_A) superimpose with 0.355 Å RMSD is shown in Figure 5, N-terminal domain has slightly higher displacement than C-terminal domain. The αC- helix in both structures is highly superimposable as shown in Figures S2A and S2B, only side chains have slightly altered conformations. Similarly the DFG motif is in inactive conformation with only slightly altered conformations (Figure S2C). These features are indicative of similar BCR-ABL kinase structure in binding to both inhibitors.

The structure superposition of ponatinib and imatinib is shown in Figure 4 and both inhibitors bind BCR-ABL kinase at exactly the same location. Further despite the peptide bond swap, both inhibitors vary only in two locations. 1. The presence of CF_3_ group on piperazine substituted phenyl ring. 2. The presence of acetylene linked imidazo [1,2b] pyridazine ring. The CF_3_ group makes close contacts with hydrophobic side chains of Leu298 and Leu354. From Table 2, we observed that Leu298 has stable interactions with CF_3_ throughout MD simulations in all mutations, while Leu354 experiences increased vdW interactions with mutations E255K and T315A. Thr315 is close to acetylene link of ponatinib and imidazo [1,2b] pyridazine ring is in a hydrophobic cavity that is enclosed by Leu248, Tyr253, Phe382, Phe317 and Leu370. Among these, Leu370 makes stable CH-pi interactions with imidazo [1,2b] pyridazine ring contributing to the stability of native and mutant complexes. The other four residues (Leu248, Tyr253, Phe382 and Phe317) in most BCR-ABL kinase mutants when bound to ponatinib undergo high conformational changes during MD simulations. We believe that these conformational changes are responsible for ponatinib binding and inhibition of native and mutant BCR-ABL kinases.

## Conclusions

The pan-BCR-ABL kinase inhibitor, ponatinib is most popular for its inhibition of ABL^T315I^ mutation at nano molar concentrations. Fourteen mutant ABL kinase structures complexed with ponatinib were modeled and we performed 25 ns of MD simulations to study the structural changes of protein when complexed with ponatinib within its binding site. Using the SIE method, we calculated binding free energies and its component of non bonding energies such as intermolecular vdW energies and reaction field energies. Further, coulomb and vdW contributions from individual amino acid residues in active site were calculated. The calculated SIE values are in the range −10.03 kcal/mol to −10.67 kcal/mol and correspond with the narrow range of IC50 values (0.5 nM to 36 nM) of native and mutant BCR-ABL kinase inhibition by ponatinib. From these MD simulations, we observed that fluctuations in residues from P-loop, β3-, β5- strands and αC- helix are mainly responsible for ponatinib binding to native and all mutant BCR-ABL kinases. Further, amino acid residues Met244, Lys245, Gln252, Gly254, Leu370 and Leu298 did not undergo any conformational changes due to mutations. The rest of the mutations effect ponatinib binding free energy calculations with its component energies evidently correlating with their activities. These studies explain the atomistic details of ponatinib binding to native and mutant BCR-ABL kinases and the results will be helpful in future modifications of ponatinib and binding calculations of new mutant ABL kinase or new inhibitors.

## Supporting Information

Figure S1 **Number of hydrogen bonds formed between ponatinib- native and mutant BCR-ABL kinases during 25 ns of simulations.**
(DOC)

Figure S2 The alignment of the C-alpha helix from imatinib bound BCR-ABL PDB_ID:1IEP (magenta) and Ponatinib bound BCR_ABL PDB_ID:3OXZ (Cyan) (A). The side chains orientations of the both C-alpha helices (B). DFG motif side chain conformations in both imatinib and ponatinib bound BCR-ABL kinase (C).
(DOC)
