# Supplementary material for: Ponatinib Is a Pan-BCR-ABL Kinase Inhibitor: MD Simulations and SIE Study
Source: PLoS One. 2013 Nov 13;8(11):e78556. doi: 10.1371/journal.pone.0078556 (PMC3827254; doi:10.1371/journal.pone.0078556)
Supplement: Figure S1 — Number of hydrogen bonds formed between ponatinib- native and mutant BCR-ABL kinases during 25 ns of simulations. (DOC) [file pone.0078556.s001.doc]

Supplementary Figure 1. Number of hydrogen bonds formed between ponatinib- native and mutant BCR-ABL kinases during 25 ns of simulations.

Time (ps)


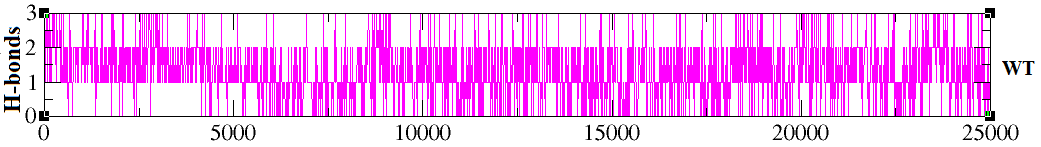

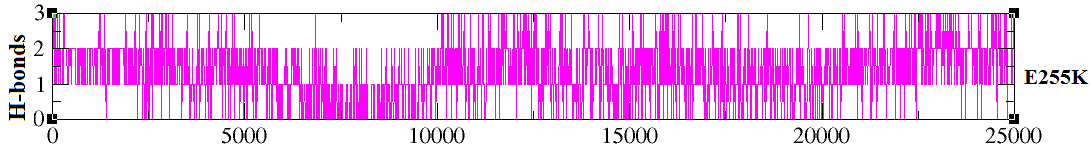

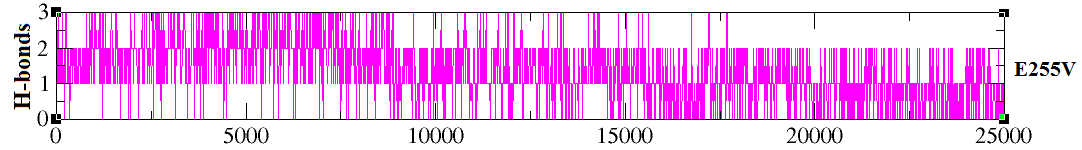

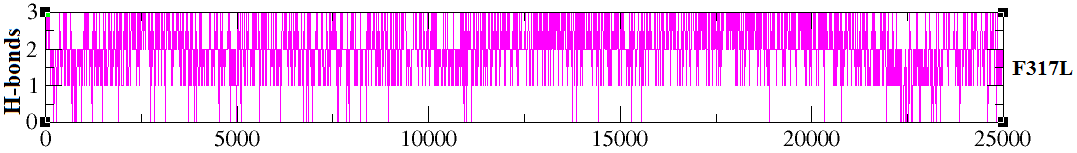

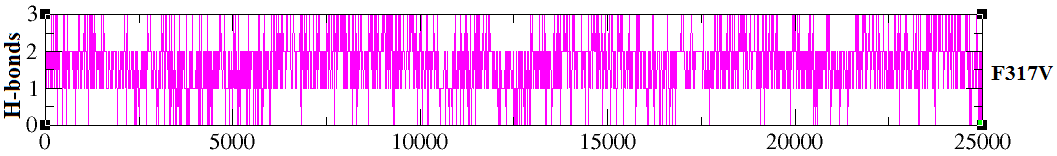

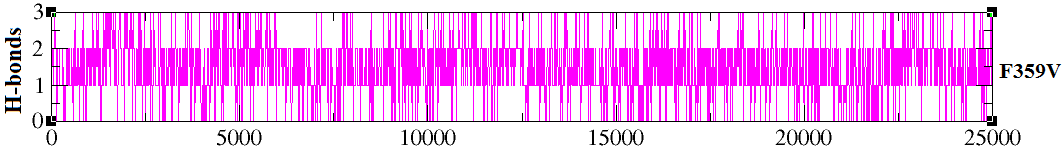

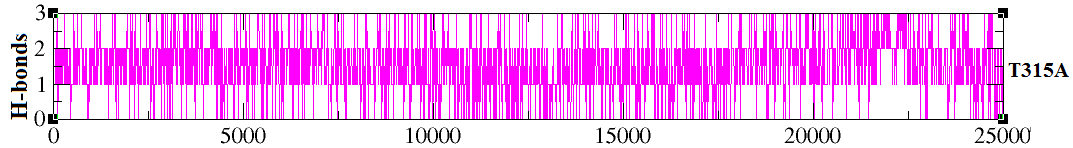

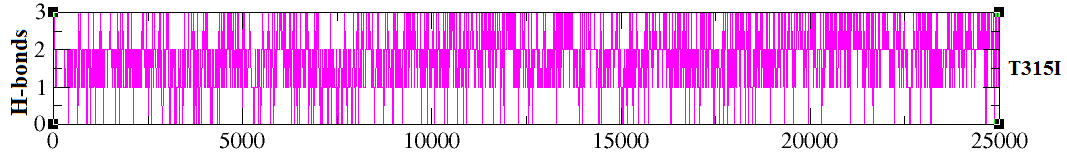

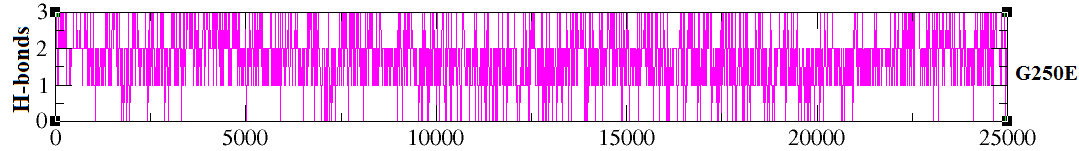

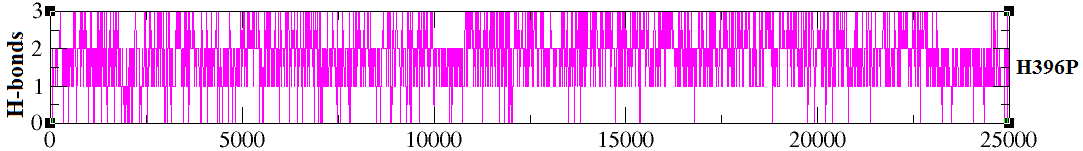

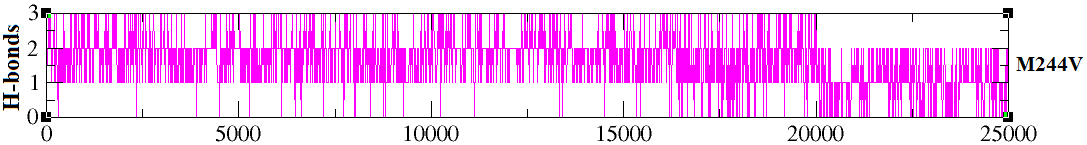

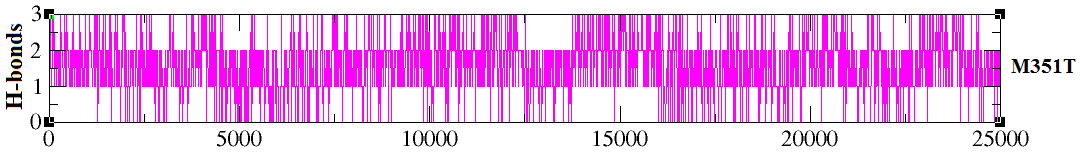

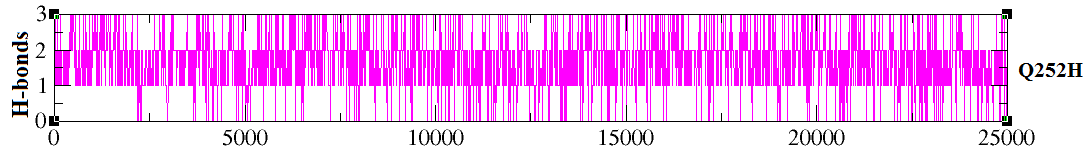

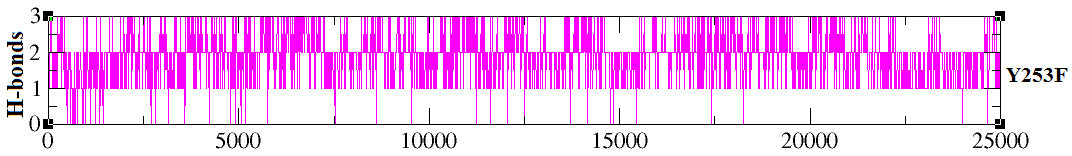

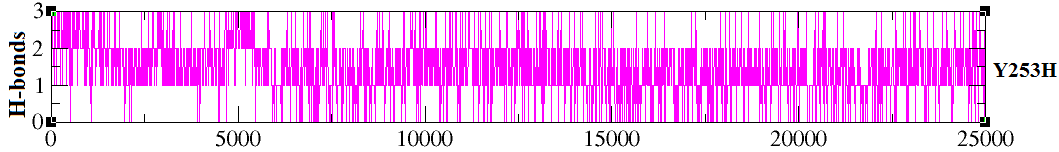


Time (ps)
