# Supplementary material for: Ponatinib Is a Pan-BCR-ABL Kinase Inhibitor: MD Simulations and SIE Study
Source: PLoS One. 2013 Nov 13;8(11):e78556. doi: 10.1371/journal.pone.0078556 (PMC3827254; doi:10.1371/journal.pone.0078556)
Supplement: Figure S2 — The alignment of the C-alpha helix from imatinib bound BCR-ABL PDB_ID:1IEP (magenta) and Ponatinib bound BCR_ABL PDB_ID:3OXZ (Cyan) (A). The side chains orientations of the both C-alpha helices (B). DFG motif side chain conformations in both imatinib and ponatinib bound BCR-ABL kinase (C). (DOC) [file pone.0078556.s002.doc]

Figure S2: A) The alignment of the C-alpha helix from imatinib bound BCR-ABL PDB_ID:1IEP (magenta) and Ponatinib bound BCR_ABL PDB_ID:3OXZ (Cyan). B) The side chains orientations of the both C-alpha helices. C) DFG motif side chain conformations in both imatinib and ponatinib bound BCR-ABL kinase


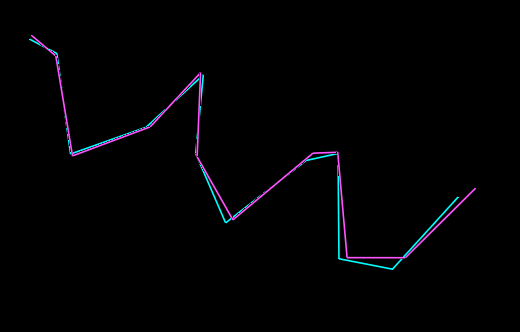


A)


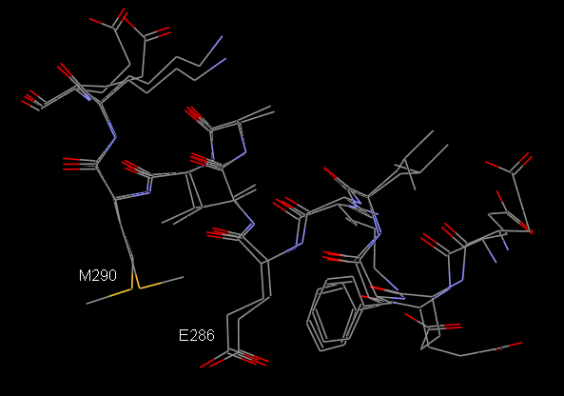


B)


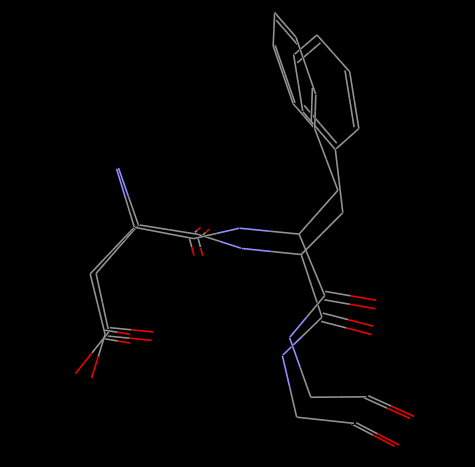


C )
